# A pretargeting system for tumor PET imaging and radioimmunotherapy

**DOI:** 10.3389/fphar.2015.00054

**Published:** 2015-03-31

**Authors:** Françoise Kraeber-Bodéré, Caroline Rousseau, Caroline Bodet-Milin, Eric Frampas, Alain Faivre-Chauvet, Aurore Rauscher, Robert M. Sharkey, David M. Goldenberg, Jean-François Chatal, Jacques Barbet

**Affiliations:** ^1^Nuclear Medicine Department, Nantes University HospitalNantes, France; ^2^Nuclear Medicine Department, Institut de Cancérologie de l'Ouest René GauducheauNantes, France; ^3^Cancer Research Center, University of Nantes, Institut National de la Santé et de la Recherche Médicale, Centre National de la Recherche ScientifiqueNantes, France; ^4^Radiology Department, Nantes University HospitalNantes, France; ^5^Immunomedics, Inc.Morris Plains, NJ, USA; ^6^Garden State Cancer Center, Center for Molecular Medicine and ImmunologyMorris Plains, NJ, USA; ^7^GIP ArronaxSaint-Herblain, France

**Keywords:** pretargeting, immunoscintigraphy, immuno-PET, radioimmunotherapy, bispecific antibody

## Abstract

Labeled antibodies, as well as their fragments and antibody-derived recombinant constructs, have long been proposed as general vectors to target radionuclides to tumor lesions for imaging and therapy. They have indeed shown promise in both imaging and therapeutic applications, but they have not fulfilled the original expectations of achieving sufficient image contrast for tumor detection or sufficient radiation dose delivered to tumors for therapy. Pretargeting was originally developed for tumor immunoscintigraphy. It was assumed that directly-radiolabled antibodies could be replaced by an unlabeled immunoconjugate capable of binding both a tumor-specific antigen and a small molecular weight molecule. The small molecular weight molecule would carry the radioactive payload and would be injected after the bispecific immunoconjugate. It has been demonstrated that this approach does allow for both antibody-specific recognition and fast clearance of the radioactive molecule, thus resulting in improved tumor-to-normal tissue contrast ratios. It was subsequently shown that pretargeting also held promise for tumor therapy, translating improved tumor-to-normal tissue contrast ratios into more specific delivery of absorbed radiation doses. Many technical approaches have been proposed to implement pretargeting, and two have been extensively documented. One is based on the avidin-biotin system, and the other on bispecific antibodies binding a tumor-specific antigen and a hapten. Both have been studied in preclinical models, as well as in several clinical studies, and have shown improved targeting efficiency. This article reviews the historical and recent preclinical and clinical advances in the use of bispecific-antibody-based pretargeting for radioimmunodetection and radioimmunotherapy of cancer. The results of recent evaluation of pretargeting in PET imaging also are discussed.

## Introduction

The idea of using antibodies to specifically deliver toxic agents to tumor cells emerged a long time ago, and was subsequently proposed to target radionuclides for tumor detection and imaging (Goldenberg et al., 1974). The concept was revived after the introduction of monoclonal antibodies, which made the development of immunoconjugates and radiolabeled compounds much easier (Kohler and Milstein, 1975). The most straightforward application was tumor imaging, since it directly showed if the result is reached: namely, was the activity concentrated on the target or not? In the mid-eighties, imaging was performed by scintigraphy with gamma emitters, such as iodine-131, indium-111, or technetium-99m. Initial results were remarkable, especially in preclinical models. Translation into humans followed, but sensitivity and specificity were not sufficient for a real commercial success, although some radiolabeled antibodies (e.g., oncoscint, CEA-Scan, prostascint, leukoscan) received market approval (Goldenberg, 1997). Thus, developing new approaches to achieve a higher tumor signal over adjacent normal tissues became a very active field of research. Goodwin and co-workers proposed the use of a specific immunoconjugate recognizing both a target antigen and a low-molecular-weight substance to which a radionuclide was bound (Goodwin et al., 1988). The unlabeled tumor-specific agent and the substance carrying the radionuclide would be injected sequentially. The rationale was that the bispecific immunoconjugate would decorate tumor cells with binding sites for the small labeled molecule, which would then bind the targets quickly with fast excretion of excess unbound activity. This approach was called pretargeting.

Pretargeting can be achieved using many different techniques (Goldenberg et al., 2006). Antibodies or antibody fragments usually provide specific binding to target tumors, but different substances have been proposed for the second recognition step. A second antibody may be used to bind a small molecule, called a hapten by immunologists, or avidin/streptavidin to bind biotin (Paganelli et al., 1991), or a nucleotide to bind a second nucleotide of complementary sequence (Bos et al., 1994). Many other receptor-ligand or enzyme-substrate systems could be used as well, and the pros and cons of these methods have been previously discussed (Goldenberg et al., 2003; Paganelli and Chinol, 2003). It is not the scope of the present review to describe all these approaches. Our focus is on reviewing antibody-based recognition systems: “bispecific” antibodies and radiolabeled haptens (Chang et al., 2002).

It became rapidly apparent that tumors not expressing the target antigen, whilst being a true-negative in molecular terms, would score as false-negatives following immunoscintigraphy. At about the same time, positron emission tomography (PET) imaging and ^18^F-fluoro-deoxy-glucose (FDG) appeared as a general, sensitive, and rather specific tumor detection method (Delbeke, 1999). Immunoscintigraphy, including pretargeted immunoscintigraphy, was soon considered obsolete. Following this it was found that pretargeting, using either the avidin/biotin or bispecific antibody techniques, was capable of delivering a high fraction of injected activity to tumors, and that tumor accretion was protracted enough to deliver high tumor absorbed doses if a therapeutic radionuclide was used for cancer therapy instead of imaging (Goldenberg et al., 2006). Thus, pretargeting became recognized as a promising approach for targeted radionuclide therapy. Even more recently, antibodies have been labeled with positron-emitters (van Dongen and Vosjan, 2010). Current improvements in antibody specificity and radiolabeling methods coupled with more sensitive and higher resolution PET imaging, makes immuno-PET a feasible approach both as a diagnostic tool or companion diagnostic, especially in the context of antibody-based cancer therapy. Pretargeting improves the performance of immuno-PET and allows for the use of short half-life positron-emitting radionuclides that would reduce patient irradiation.

The results obtained from preclinical experiments and clinical applications for both imaging and therapy will be discussed, along with recent results in pretargeted PET imaging.

## Pretargeting with bispecific antibodies and radiolabeled haptens

Radiolabeled antibodies in the form of whole IgG molecules show slow pharmacokinetics. Whilst tumor accretion may reach fairly high levels (more than 10% of injected activity per gram of tumor in mice, and sometimes as high as 0.1% per gram of tumor in humans), this is obtained only hours or days after activity injection, and circulating unbound activities remain elevated for days (Chang et al., 2002). Using alternative antibody formats, such as antibody fragments or low-molecular weight recombinant molecules, can enhance certain targeting properties, but there is an inevitable trade-off between tumor accretion and clearance of excess unbound activity (Jain et al., 2007). Fine-tuning a targeting strategy that preserves the high tumor accretion observed with directly radiolabeled IgG, whilst rapidly eliminating non-targeted radioactivity from the blood and normal tissues to increase tumor-to-normal tissue ratios and minimize toxicity has been an active research field since the mid-eighties.

As mentioned, Goodwin et al. (1988; Goldenberg, 1997) proposed the use of a specific immunoconjugate that recognizes both the target antigen and a low-molecular-weight substance to which the radionuclide is bound. The unlabeled tumor-seeking agent and the substance carrying the radionuclide are injected sequentially. The radioactive compound is administered only after the antibody has localized in the target lesion and its excess has cleared, at least in part, from the circulation. This was first achieved using a bispecific antibody binding both a target antigen and a radiometal-chelate complex (Stickney et al., 1991; Lollo et al., 1994). In the early days of pretargeting, the main objective was tumor imaging, and indium-111 was considered the radionuclide of choice. More specifically, an indium-benzyl-EDTA complex was used as a hapten and bispecific antibodies binding carcinoembryonic antigen (CEA) and the hapten were used in the preclinical and clinical settings (Stickney et al., 1991; Lollo et al., 1994). Whilst the results were encouraging, if the injected dose of bispecific antibody was low or the delay between bispecific antibody and hapten injections was too long, low tumor uptake was observed. Conversely, with a high dose of bispecific antibody and a short delay, tumor uptake was appreciable, but retention of the radioactive hapten in the circulation was high and protracted. A significant improvement was thus necessary.

## The affinity enhancement system

**Figure 1:**
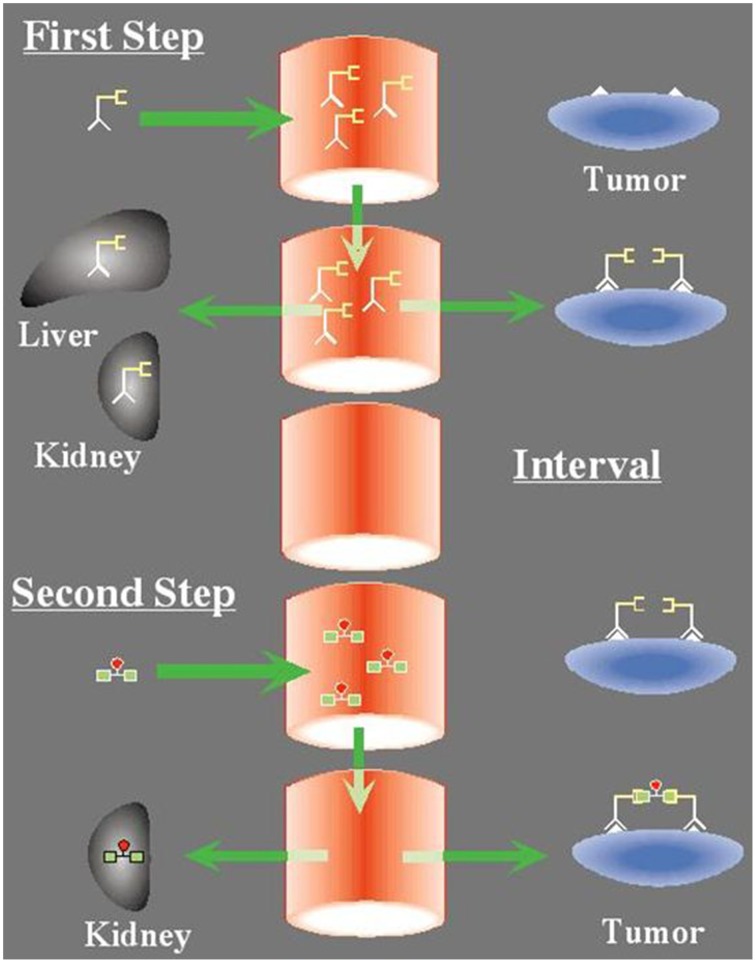
**The concept of pretargeting with the Affinity Enhancement System**. First Step: A bispecific antibody, designed to bind by one arm a tumor antigen (e.g., carcinomembryonic antigen) and by the other a hapten (e.g., the indium-DTPA complex or the HSG pseudo-peptide), is injected first. It distributes in the body and binds the tumor. Second Step: After an interval of several hours to a few days, the bispecific antibody has cleared from the circulation and the radiolabeled bivalent hapten is injected. It binds rapidly to the tumor. At the tumor cell surface, hapten bivalency induces cooperativity that results in very slow release.

The use of bivalent haptens was proposed by Le Doussal et al. (1989, 1990). The idea was that bivalence could cross-link bispecific antibody molecules at the surface of target cells, resulting in a cooperative binding and enhanced affinity (or avidity), whereas, in the circulation, binding would remain rapidly reversible. This is why this technique was called the “Affinity Enhancement System” or AES (Figure 1). It must be noted that because of the avidity effect, no chase of excess bispecific antibody is necessary with AES, and surprisingly, the distance between the two haptens does not need to be very long. For instance, the tyrosyl-lysine dipeptide, substituted by DTPA on both the α-NH_2_ of the tyrosine and the ε-NH_2_ of the lysine, allows for the simultaneous binding of two anti-DTPA-indium antibodies, as shown by *in vitro* sandwich assays (Le Doussal et al., 1990).

This concept was used by Goodwin et al., who confirmed the superiority of bivalent haptens in this context (Goodwin et al., 1992, 1994), and proposed its use for therapy using a monoclonal antibody raised against the yttrium-DOTA hapten. Later on, similar results were published that further confirmed the advantage of using bivalent haptens (Kranenborg et al., 1998; Boerman et al., 1999) using a hybrid hybridoma obtained by somatic fusion of the anti-renal cell carcinoma antibody G250 with an anti-DTPA-indium antibody. Soon, indium-111 scintigraphy imaging studies were translated to the clinic, which afforded high contrast images (Le Doussal et al., 1993; Peltier et al., 1993; Vuillez et al., 1997; Barbet et al., 1998), particularly in medullary thyroid carcinoma (MTC), a tumor that consistently expresses high levels of CEA. The bispecific antibody F6x734 recognized CEA on one side and the hapten indium-DTPA on the other. It was merely a chemical conjugate of two Fab fragments. The bivalent hapten was prepared by derivatizing tyrosyl-lysine with DTPA anhydride. This bivalent hapten could be labeled with indium-111, but also radio-iodinated. However, the antibody was specific for the indium-DTPA complex and other metal radionuclides could not be used. Bispecific antibodies and bivalent haptens based on the histidine-succinyl-glycine (HSG) pseudo-peptide were then proposed (Janevik-Ivanovska et al., 1997). New bivalent haptens based on this HSG structure that may be labeled using a variety of radionuclides are currently used for pretargeted radioimmunotherapy and immuno-PET.

## Pretargeted radioimmunotherapy (pRIT)

By the time these preliminary clinical studies of tumor imaging were completed, FDG-PET was developed for staging and therapy evaluation in solid tumors and lymphomas (Bos et al., 1994). Specific delivery of indium-111 to tumors using pretargeting was highly specific, but missed antigen-negative tumor sites or those expressing very low levels of target antigen, resulting in moderate sensitivity (Barbet et al., 1998). In contrast, FDG-PET proved to have excellent sensitivity and good specificity for many different solid tumors and aggressive lymphomas. The ability of a single agent to image most tumors and the high sensitivity and resolution of PET cameras made the technique extremely successful. Most efforts in the development of radiolabeled antibodies for scintigraphy were soon discontinued. However, preclinical studies had shown that pretargeting could compare favorably with directly labeled antibodies for therapy. Pretargeting was shown to be able to deliver tumoricidal radiation doses (Gautherot et al., 1998) and cure mice bearing human tumor xenografts (Kraeber-Bodéré et al., 1999a; Gautherot et al., 2000).

Monoclonal antibodies have been regarded for years as ideal to deliver radionuclides to disseminated tumor cells, and the most convincing results in radioimmunotherapy (RIT) have been obtained with antibodies to B-lymphocyte differentiation antigens (CD20, CD22, CD37, HLA-DR) to treat B-cell lymphomas. Most of these antibodies produce significant intrinsic anti-tumor activity (e.g., rituximab) by triggering immune defense mechanisms (Maloney et al., 1997). The accessibility of target cells to blood-borne therapy and the high radiosensitivity of lymphomas account for the observed clinical efficacy, which can be considered as a real breakthrough in the treatment of chemoresistant forms of these cancers (Wilder et al., 1996). Two anti-CD20 radiolabeled antibodies have been marketed: ^131^I-tositumomab (Bexxar®, GlaxoSmithKline) and ^90^Y-ibritumomab tiuxetan (Zevalin®, Spectrum Pharmaceuticals, Henderson, NV, USA), whilst Bexxar has been recently discontinued.

Monoclonal antibodies have also shown efficacy in the treatment of solid tumors, generally in combination with chemotherapy. Trastuzumab and other antibodies targeting growth factor receptors are now on the market. Many other antibodies with very high specificity for solid tumors are available [e.g., anti-CEA antibodies for colorectal carcinomas (CRC), MTC, breast cancers, or lung cancers]. However, poor tumor accessibility and low radiosensitivity characterize most solid tumors. As a result, clinical trials with radiolabeled antibodies to date have provided limited and contested results for solid tumor RIT. Antibodies or antibody fragments labeled with β^−^-emitting radioisotopes (iodine-131, yttrium-90, rhenium-186 or 188, copper-67) still deliver too much radiation to normal organs compared to tumor-specific exposure (Wilder et al., 1996).

In these cases, pretargeted RIT could increase the therapeutic index as it increases tumor-to-normal tissue uptake ratios, and increases radiation doses delivered to tumor cells in the clinic, as it does in preclinical models. A phase I/II clinical trial was implemented in 1996 to evaluate toxicity, pharmacokinetics, dosimetry, and antitumor activity, using the murine anti-CEA bispecific antibody F6x734 and the bivalent indium-DTPA hapten labeled with iodine-131 in 26 patients with recurrence of MTC (Kraeber-Bodéré et al., 1999b). Hematotoxicity was the dose-limiting toxicity. The maximum tolerated activity was relatively low (1.8 GBq/m^2^), because diffuse bone marrow involvement appeared to be more frequent than previously reported in patients with metastatic MTC (Mirallié et al., 2005). Some therapeutic response was observed, and was mainly in patients with a small tumor burden and after repeated courses of pretargeted RIT. Because of frequent immune responses, a chimeric (hMN14x734) bispecific antibody was developed. A prospective phase I optimization study was performed in 34 patients with CEA-expressing tumors, including MTC and other carcinomas, to determine the optimal bispecific antibody dose, hapten activity, and pretargeting interval (Kraeber-Bodéré et al., 2003, 2006).

A major and rather unexpected outcome of these studies was the high number of patients with long-term disease stabilization, which was observed in 53% of the MTC patients based on morphological imaging assessment (computed tomography, MRI) and biomarker evaluation using serial calcitonin (Ct) and CEA measurements. A retrospective study compared the overall survival (OS) of 29 MTC patients treated by pretargeted RIT in the 2 phase I/II trials previously presented, against 39 contemporaneous untreated patients for whom data were collected by the French Endocrine Tumor Group (GTE) (Chatal et al., 2006). Since Ct and CEA doubling times (DT) had been shown to be of a prognostic value for survival in MTC (Barbet et al., 2005), patients were stratified accordingly; OS was significantly longer in high-risk (Ct DT < 2 years) treated compared to high-risk untreated patients (median OS, 110 vs. 61 months; *P* < 0.030). Ct DT variations were proposed as a surrogate marker for survival by comparing, among treated patients, the survival of biological responders and non-responders. Defining a responder as showing at least a 100% increase (doubling) in Ct DT, 47% of biological responders experienced significantly longer survival than non-responders (median OS, 159 vs. 109 months; *P* < 0.035) or untreated patients (median OS, 159 vs. 61 months; *P* < 0.010). Treated patients with bone/bone marrow disease had a longer survival than patients without such involvement (10-year OS of 83 vs. 14%; *P* < 0.023). Toxicity was mainly hematological and related to bone marrow involvement, and was treated by platelet infusions or G-CSF injections. Interestingly, no other toxicity, especially renal toxicity, was reported.

A prospective phase II pretargeted RIT trial was undertaken in progressive MTC patients, progression being defined biologically by a Ct DT shorter than 5 years (Salaun et al., 2012). Forty-five patients were enrolled. Three patients did not complete the treatment, two due to reactions during bispecific antibody infusion, and hapten-labeling failure in one. Patients received 40 mg/m^2^ of hMN-14x734, followed by 1.8 GBq/m^2^ of bivalent indium-DTPA hapten labeled with iodine-131 given 4–6 days later. According to RECIST morphological imaging criteria, the disease control rate (durable stabilization plus objective response) was 76.2%, including a durable complete response of at least 40 months in one patient (2.4%), and durable stable disease (≥6 months) in 31 patients (73.8%). Tumor uptake assessed by post-pretargeted RIT immunoscintigraphy predicted significantly the tumor response. After RIT, 21 of 37 assessed patients (56.7%) showed a ≥100% increase in Ct or CEA DT or prolonged decrease of the biomarker concentration. Interestingly, biomarker DT and FDG-PET response after pretargeted RIT showed a prognostic value for OS, suggesting these data might serve as surrogate markers for prognostically favorable patients (Salaun et al., 2014). As expected, in the patients with a high frequency of diffuse bone marrow involvement, grade 3 and 4 hematologic toxicity was observed (54.7% of patients), and myelodysplastic syndrome was reported in two cases, including one previously heavily treated patient.

## Current research on pretargeting

The use of indium-DTPA as a hapten for pretargeting is known to be problematic, because labeling is only possible with indium-111 or iodine-123 for scintigraphy, iodine-124 for PET or iodine-131 for therapy. Interest therefore switched to another hapten, histamine-succinyl-glycine (HSG). Several different bivalent structures were developed with a view to make labeling possible with a variety of radionuclides. A bivalent HSG hapten that could be labeled with technetium-99m or rhenium-186/188 was produced first (Gestin et al., 2001). Subsequently, a series of bivalent HSG haptens was synthesized by Goldenberg and coworkers that could be labeled with a variety of radionuclides (Sharkey et al., 2003; McBride et al., 2006). One of them, IMP 288, was used in many preclinical and clinical studies. Labeling with iodine-124 and gallium-68 was used for immuno-PET and a method to label this peptide with fluorine-18 was also described (McBride et al., 2009; Schoffelen et al., 2010a). For therapy, IMP 288 was labeled with yttrium-90 (Karacay et al., 2009) and lutetium-177 (Schoffelen et al., 2010b).

To further advance the clinical applications of pretargeting, it also became clear that humanized recombinant bispecific antibodies must be produced. Although many different formats of bispecific antibodies had been proposed, mass production under GMP conditions proved quite difficult. Eventually, Immunomedics, Inc., developed a new approach they refer to as the Dock-and-Lock™ (DNL) method (Rossi et al., 2006). The DNL method uses the regulatory subunits of cAMP-dependent protein kinase and the anchoring domains of A kinase to construct conjugates of antibody Fab fragments that are bispecific and trivalent, with a single binding site for the hapten and two binding sites for the tumor antigen. They have successfully produced several DNL™ conjugates binding CEA (TF2), CD20 (TF4), a mucin antigen expressed by pancreatic tumors (TF10), and the Trop-2 antigen (TF12) (Govindan and Goldenberg, 2010; Sharkey et al., 2012). Through these remarkable achievements, using this technology, radionuclide pretargeting is now possible against a wide spectrum of target antigens, and allows a variety of radionuclides for PET imaging and therapy to be mobilized (Karacay et al., 2009; Schoffelen et al., 2010b, 2012; van Rij et al., 2013). The first clinical results of an optimization study assessing the anti-CEA × anti-HSG bsMAb TF2 and the radiolabeled hapten-peptide, ^177^Lu-IMP288, in patients with metastatic CRC have been reported recently (Schoffelen et al., 2013). Different schedules were studied in four cohorts of five patients: (1) shortening the interval between the bsMAb and peptide administration (5 days to 1 day), (2) escalating the TF2 dose (from 75 to 150 mg), and (3) reducing the peptide dose (from 100 to 25 μg). Rapid and selective tumor uptake was detected within 1 h after the peptide injection, with high tumor-to-tissue ratios at 24 h. The best tumor targeting was achieved with a 1-day pretargeting interval and with the 25-μg peptide dose. High activities of ^177^Lu-IMP288 (2.5–7.4 GBq) were well-tolerated, with some manageable reactions during the TF2 infusions, and transient grade 3–4 thrombocytopenia in 10% of the patients. Dosimetry estimations concluded that renal and red bone marrow uptake of ^177^Lu-IMP288 peptide was relatively low, even if marrow doses increased in subsequent cohorts as the TF2/Lu-IMP288 ratio was increased (Schoffelen et al., 2014). The predicted kidney absorbed doses (<0.50 mGy/MBq) did not limit the maximum activity that could be administered. None of the patients exceeded the limit of 15 Gy to the kidneys with four cycles of 7.4 GBq ^177^Lu-IMP288. Two phase-I clinical trials are on-going in France, assessing pretargeted ^177^Lu-IMP288 (one injection) in patients with metastatic CEA-positive lung carcinoma, and fractionated injection of ^90^Y-IMP288 in metastatic CRC patients.

Preclinical pharmacokinetic and dosimetry studies have shown that high radiation doses could be delivered to tumor cells, with limited irradiation of normal tissues (Frampas et al., 2011). These studies have also demonstrated that pretargeting using DNL bispecific antibodies would be most useful for delivering short half-life radionuclides. Indeed, the fast tumor uptake of activity afforded by this pretargeting technique would be suitable for radionuclides with half-lives of a few hours. Since all pretargeting optimization studies using the DNL bispecific antibodies have shown that a large molar excess of bispecific antibody relative to the molar amount of hapten should be used, a potentially limiting factor is the specific activity at which the bivalent haptens (e.g., IMP 288) may be labeled, which is also, at least theoretically, in favor of short half-life radionuclides. In preclinical studies with lutetium-177, the limited specific activity was overcome by using repeated injections of both the bispecific antibody and IMP 288 labeled with lutetium-177 (van Rij et al., 2013). Also, yttrium-90 outperformed lutetium-177 in this context, and short half-life alpha-particle-emitting radionuclides, such as astatine-211 or bismuth-213, should be considered (Frampas et al., 2011).

## Pretargeted immuno-PET

The ability of pretargeting to deliver activity within a few hours and to achieve high tumor-to-background activity ratios revived the idea of antibody-targeted *in vivo* imaging. Obviously a most attractive approach in this context would be PET, and the major justification for antibody-targeted *in vivo* imaging would be to select patients who could benefit from antibody-targeted therapy, because their tumors express the target antigen. This would replace tumor biopsy, which obviously cannot be performed for all known tumor lesions and is not fully accurate because a biopsy may not be representative of the whole tumor.

**Figure 2:**
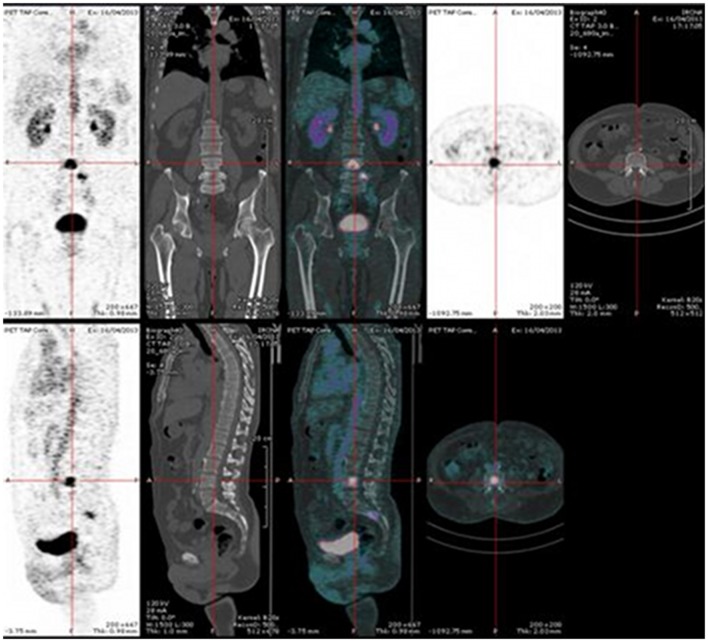
**Pretargeted immuno-PET recorded after injection of 120 nmol of TF2 and 6 nmol of ^68^Ga-IMP-288 at 30 h in a patient with a relapse of MTC**. Immuno-PET shows bone marrow lesions.

**Figure 3:**
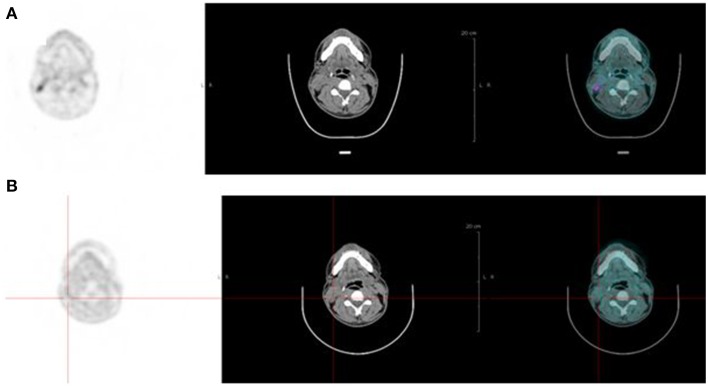
**Pretargeted immuno-PET recorded after injection of 120 nmol of TF2 and 6 nmol of ^68^Ga-IMP-288 at 42 h in a patient with a relapse of MTC**. Immuno-PET **(A)** shows right neck lymph nodes not detected by F-DOPA-PET **(B)**.

**Figure 4:**
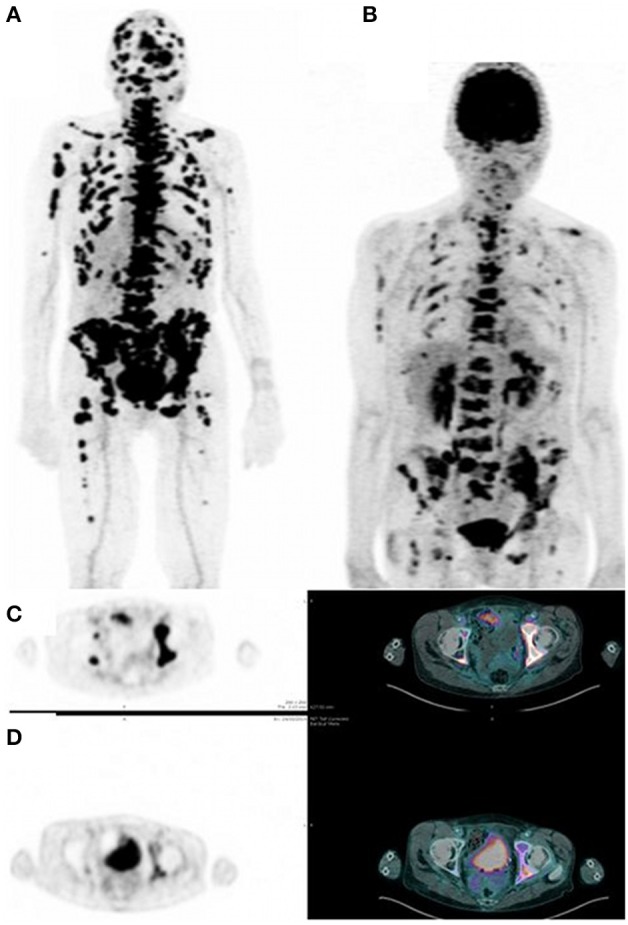
**Pretargeted immuno-PET recorded after injection of 120 nmol of TF2 and 3 nmol of ^68^Ga-IMP-288 at 30 h in a patient with metastatic breast carcinoma**. Immuno-PET **(A,C)** shows a high tumor uptake and a higher number of lesions as compared to FDG-PET **(B,D)**.

PET, with its improved sensitivity and better resolution, as compared to scintigraphy and SPECT in humans, is indeed expected to provide useful clinical information. PET imaging with IgG antibodies labeled with long-lived positron-emitting radionuclides, such as zirconium-89, has been shown to be quite effective for *in vivo* tumor detection and to demonstrate antigen expression (Vugts et al., 2013). However, PET must be recorded days after activity injection for optimal contrast, and these long-lived radionuclides that emit, in addition to a positron, gamma rays of high energy have an unfavorable dosimetry. Pretargeting makes possible the use of short-lived radionuclides, such as gallium-68, but also fluorine-18. The PET images may be recorded only a few hours after activity injection. This has already been shown in preclinical studies (McBride et al., 2006; Schoffelen et al., 2010a, 2012), and clinical trials are in progress that show very promising sensitivity and specificity (Schuhmacher et al., 2001). Two clinical trials are on-going in France, aiming at optimizing and assessing pretargeted immuno-PET, using anti-CEA TF2 and ^68^Ga-IMP288 in patients with relapsed MTC and HER2-negative breast cancer. In each study, four or five cohorts of three patients are compared to determine the best TF2/IMP-288 molar dose ratio and the best pretargeted delay. Figure 2 illustrates the high tumor uptake obtained with this approach in a MTC patient with bone marrow lesions, injected with 120 nmol of TF2 and 6 nmol of ^68^Ga-IMP-288 30 h later. In some cases, immuno-PET allowed detection of lesions not detected by F-DOPA-PET, considered as reference PET imaging in MTC (Figure 3). Promising results were also obtained in a CEA-positive breast cancer patients (Figure 4).

## Conclusions and perspectives

Pretargeting is an old idea originating in the mid-80s. Several approaches have been developed and tested in preclinical models and clinical trials. Here, approaches that use bispecific antibodies targeting a tumor antigen and a hapten have been discussed and the superiority of bivalent haptens has been stressed. Originally, pretargeting was developed for *in vivo* imaging using single-photon scintigraphy until FDG-PET become so popular because it demonstrated such high sensitivity in a large number of tumors, while antibody-targeted radionuclides could only target tumors expressing specific antigens. Following this, pretargeting was also considered for radionuclide therapy. Phase I and II clinical studies demonstrated the efficacy of anti-CEA pretargeting in rapidly progressive metastatic MTC patients, paving the way for future studies.

Today, a new generation of pretargeting compounds are available. A series of humanized recombinant trivalent bispecific antibodies based on DNL technology have been produced by Goldenberg and coworkers. They recognize antigens of high interest, including CEA, CD20, Trop-2, and the hapten peptide, HSG, which may easily be labeled with a large variety of radionuclides (Sharkey et al., 2003; McBride et al., 2006). They may be produced in large amounts and may be used in the clinic for both imaging and therapy.

Several preclinical studies of pretargeted radionuclide therapy using these new compounds have produced promising results and clinical trials are in progress. The very fast delivery of radionuclides using this pretargeting approach makes it possible to develop immuno-PET with short half-life positron-emitters. This has been established in preclinical studies and clinical trials are in progress, confirming that high quality images can be obtained.

### Conflict of interest statement

Dr. Sharkey is an employee of Immunomedics, Inc. Dr. Goldenberg is stockholder and officer of Immunomedics, Inc. The other authors declare that the research was conducted in the absence of any commercial or financial relationships that could be construed as a potential conflict of interest.

## Abbreviations

CEA: carcinoembryonic antigen
CRC: colorectal carcinomas
Ct: calcitonin
DNL: Dock-and-Lock
DT: doubling times
DTPA: diethylene triamine pentaacetic acid
FDG: 18 F-fluoro-deoxy-glucose
F-DOPA: L-3,4-Dihydroxy-6-[18 F]fluorophenylalanine
HSG: histidine-succinyl-glycine
MTC: medullary thyroid carcinoma
OS: overall survival
PET: positron emission tomography
RIT: radioimmunotherapy
SPECT: single photon emission tomography.
